# Culturally sensitive active ageing seen through the lens of the welfare theory of health: assistant nurses’ views

**DOI:** 10.3389/fpsyg.2023.1161688

**Published:** 2023-09-11

**Authors:** Carl Johansson, Lena-Karin Gustafsson, Daniel Lindberg, Ildikó Asztalos Morell

**Affiliations:** ^1^School of Health, Care and Social Welfare, Mälardalen University, Eskilstuna, Sweden; ^2^School of Urban and Rural Development, Swedish University of Agriculture, Uppsala, Sweden

**Keywords:** assistant nurses, care, good ageing, older adults, resources, vital life goals, cultural sensitivity, active ageing

## Abstract

Assistant nurses caring for older adults with immigrant backgrounds are on the front lines of a practical, theoretical, and policy battlefield. They need to implement culturally sensitive care provision while not overstating the importance of culture, thereby, contributing to a negative picture of older immigrants as especially problematic. One proposed way to strike such a balance is the welfare theory of health (WTH). In this article, we let assistant nurses apply the WTH to a series of questions in four different vignettes representing the life stories of older persons who characterize typical dilemmas described by the theory. The results show that, through the lens of the WTH, assistant nurses looked for individual care preferences rather than stereotypical ideas about cultural characteristics. Further, the assistant nurses expressed a desire to get to know the persons more deeply to better interpret and understand their individual preferences. Thus, the theoretical framework is useful not only for exposing vulnerabilities to which some older adults with immigrant backgrounds may be exposed, but also for finding ways to mitigate the vulnerability by illuminating vital life goals and using them as a framework to organize care. This approach allows for mitigating the gap between the vital life goals and available resources to achieve a holistic state of health.

## 1. Introduction

This study addresses the issue of culturally sensitive elderly care in Sweden and how it can be implemented in a way that reduces the stereotyping of immigrants. In recent years immigration to Sweden has increased, especially due to the civil war in Syria and other emergencies across the globe. The situation has had a major impact on the political discourse on immigration policies in Sweden, setting the country on a more restrictive course (Hagelund, 2020). Although access to Sweden has become increasingly limited, especially for family members hoping for reunification with next of kin who have been granted asylum (SFS, 2016, p. 752), many older adults have already taken up residence in Sweden. In 2019, 13% of the older adults above the national retirement age (65) were of foreign background (Statistics Sweden, n.d.). Sweden has a long tradition of providing culturally sensitive care solutions for older adults with minority backgrounds. This is often presented as a solution to the challenges that older adults with an immigrant background are believed to pose for the welfare state (Forssell and Torres, 2012). Applying cultural sensitivity to elderly care has been a cherished goal of policymakers (National Board of Health and Welfare, 2020) and non-governmental organizations representing minority groups (Sasson, 2001; Reidun, 2010). It also meshes with the universalism and pluralism that have long been hallmarks of Swedish welfare policy (Szebehely and Meagher, 2018). The professional role of welfare workers in Sweden (and Europe) is closely intertwined with policymaking, as the state plays an active role in supplying both the framework and the material (Brante, 2013). In recent years, Sweden’s welfare policies have taken a neo-liberal turn (Herz, 2012; Andersson and Kvist, 2015; Burstrom, 2015). The doctrine of new public management gained ground (Green-Pedersen, 2002; Levay and Waks, 2009), advocating evidence-based practice in social services (Denvall, 2008) and promoting their commodification. An alternative way of measuring quality in welfare interventions has also developed, based on the idea that the recipients are customers who can simply change suppliers if they are unsatisfied.

Culturally sensitive care has traditionally been studied using ethnicity as a social category, directing attention at various immigrant groups, their specific cultural needs and how welfare providers can meet these needs (Lill, 2017). A smaller amount of research has viewed ethnicity as a social position, which would more naturally emphasize ethnicity’s complexity as a social process that evolves, rather than a fixed category. Such studies have pointed to the importance that care providers and recipients have a shared language to reduce the risk of care recipients being misunderstood and even misdiagnosed (Jones and van Amelsvoort Jones, 1986; Ekman et al., 1995; Bischoff et al., 2003), the importance of familiar environments for people with dementia, and the fact that older immigrant adults have an increased risk of losing a language acquired in adulthood when suffering from dementia or stroke, further increasing the importance of welfare workers with diverse linguistic skills (Hyltestam and Obler, 1989; Ekman et al., 1995). Thus, cultural sensitivity to language and environmental factors has been described as an important skill set in caring for older adults with immigrant backgrounds. Torres (2006) has criticized that older adults with immigrant backgrounds are expected to have cultural needs that pose a problem for care providers to satisfy. Torres argues that such an approach fuels the psychological process of othering, whereby people seek ways to categorize people to make them understandable. This process allows us to ascribe positive attributes to categories of people that are closer to our categories, and negative attributes to others. If unchecked, the process of othering can lead to stereotyping and racism. Also, Tervalon and Murray-García (1998) argue that a professional’s inability to understand “the other” is dangerous since decisions based on fixed categories in caring environments disadvantage persons of minority cultures. Tervalon and Murray-García propose that care professionals should be trained in cultural humility, a practice that emphasises self-reflexivity and patient-focused interviews that aims to understand the individual patient and not the patient as representative of a minority group. Thus, a call for cultural sensitivity has emerged that aligns with the demands of policymakers, NGOs, and the wishes of researchers calling for awareness of othering.

Figuring out which parts of the care are culturally influenced and which are not is no easy task, partly because culture and ethnicity are so easily conflated, and also because the meaning of cultural sensitivity changes over time and across space. For example, Ismail (2021) has shown how the interpretation of religiously appropriate care may change and be interpreted differently within a single family. Recent research has abandoned the use of cultural and ethnic background as a variable to describe the perceived special needs of particular groups. Instead, the focus has turned toward what culture and ethnicity mean in the time and space shared by a care recipient and a caregiver. Lill (2017) has shown that ideas about cultural and ethnic characteristics held by professionals in elderly care impact how the work is organized and ethnicity is continuously enacted. How ethnicity is talked about, described, and observed matters for how elderly care staff treat each other and their clients based on ethnicity. Because ethnicity is constantly changing, depending on ethnic relations, different needs (social, practical, and medical) may be misinterpreted by care staff as cultural preferences (Lill and Rämgård, 2016). Care staff have been reported to feel better when given the tools to conduct an intersectional power analysis in their work (Cuesta and Rämgård, 2016). Communication and activities are two interpretations of cultural sensitivity that are often emphasized by care personnel (Söderman and Rosendahl, 2016). The participation of next of kin in someone’s daily care is seen as a resource to adapt the care to align as much as possible with the perceived cultural preferences of the person (Rosendahl et al., 2016). The ethnicity of care workers has been seen as an important factor in responding to older adult care recipients’ cultural needs and expectations. Lill (2010) argues that although it is important for older adults with immigrant backgrounds to communicate well with their care staff, this may also cause problems for the staff. Becoming the line of communication between the service provider and the older adult and his or her family may wear down the professionals, making it difficult for them to say no to the family when they ask for something extra or to give the other care recipients the attention they need.

We do not wish to disregard the importance of prior studies or more recent approaches to cultural sensitivity in elderly care. These all describe the needs of older adults, NGOs, policymakers, and researchers. We rather draw on them to suggest a new theoretical framework for approaching cultural sensitivity within Swedish elderly care. One way of handling this complexity, which has been proposed by Johansson et al. (2021), is based on the so-called vital life goals (VLGs) of older adults. VLGs are a central part of the so-called welfare theory of health (Nordenfelt, 1993), which claims that health should be regarded as a person’s ability to reach a minimal level of happiness.

If culturally influenced activities or preferences lie within the scope of such happiness, they can be focused upon by professionals. Johansson et al. (2021) argue that this approach to cultural sensitivity not only lets the older adult herself guide what should be counted as culturally important, but also has the potential to reveal inequalities when older adults’ life situations are regarded as facilitating or inhibiting cultural sensitivity. They asked older adults with immigrant backgrounds about their VLGs and how they would prefer to reach them. The results gave a nuanced image of (1) the VLGs themselves, often reported to be close to family and family-provided care, (2) their resources/possibilities to access such care, and (3) and how the core values of the reported VLGs could be accessed even if formal family-provided care could not be offered. The agency gap that Johansson et al. describe consists of an imagined ageing rooted in norms from a non-Swedish context. Such ageing is typically related to close family ties and being cared for by family and/or friends. Such care arrangements are, to some extent, controversial in Sweden because of how welfare provision is financed and organized (Szebehely and Meagher, 2018) and because it collides with strongly held ideals of gender equality and pluralism (Forssell et al., 2014). Thus, support for such arrangements is rarely obtainable in Sweden. The authors described four types of agency gaps: (1) those who want to be cared for by their immediate family, and families that also want to care for the older adult but cannot afford to take time off from work that such an assignment would require; (2) those who prefer to combine care from friends and neighbors with formal municipal care, (3) those who prefer to be cared for by ethnic organizations, and (4) those who have not had time to accumulate enough social capital to enable any of the three previous types of care. These older adults are in an acute state of agency gap, as they do not have the ability to call upon friends, family or NGOs. Yet they still feel mistrust toward municipal elderly care.

Thus, the WTH helped illuminate both older adults’ VLGs and their resources to reach the goals, expressed as social capital. This approach allowed the researchers to reveal a need for cultural sensitivity in elderly care while also avoiding the danger of explaining the preferences of older adults with immigrant backgrounds as primarily due to culture. This study builds on the results of Johansson et al. (2021) that describe the WTH as a way to approach the personal preferences of older adults expressed as VLGs, the resources a person has to reach their VLGs, and the agency gap between the VLGs and individual resources. This study aims to illuminate how the WTH can be used by professionals within elderly care as a foundation for managing the agency gap in achieving culturally sensitive care. The research questions are as follows. (1) How do professionals perceive the vulnerabilities of older adults? (understood as an agency gap between older adults’ desired VLGs and their abilities to reach them). (2) How do professionals perceive their own scope of action to work with VLGs as a tool to assist older adults?

## 2. Methodology

We make use of the vignette method with a qualitative approach to frame four cases based on the findings of our earlier interview study (Johansson et al., 2021). The vignette method is especially suitable when the topic of inquiry is sensitive. In this case, it makes it easier to study informants’ approaches to cultural sensitivity because they can reason around a fictional case without reporting what they have done in reality (Wilks, 2004; Brunnberg and Kullberg, 2013). This also adds to the reliability of the study by reducing informants’ desire to give socially acceptable answers. The method is also considered suitable to uncover professionals’ conceptions, explanations, norms, values, and ethics (Hebert et al., 1990; Lester et al., 1991) as well as attitudes (Dale and Middleton, 1990; Groskind, 1991), assessments and associations (West et al., 1984). All these aspects lie within the scope of interest of this study.

### 2.1. Sample

The informants work as assistant nurses in municipal home care organizations in the Mälardalen area, Sweden. The assistant nurses were contacted through a research collaboration between Mälardalen University and two medium-sized partnership municipalities in Sweden. Ten informants agreed to participate in the study, while 37 declined. The informants in this study were nine females and one male. They ranged from 37 to 67 years old and had various experiences working as assistant nurses in elderly care, ranging from one to 40 years of experience. All were trained assistant nurses working in municipal home care organizations. The informants work in organizations that have previously participated in projects to promote reablement, a process grounded in the WTH where an interprofessional team deliver short-term and goal-directed interventions to support older adults to return to their daily life as soon as possible after a period in hospital (Gustafsson et al., 2019). The informants’ previous knowledge about the theory is, however, unknown.

### 2.2. Design, content, and use of vignettes

The vignettes used in this study are based on the knowledge generated from the work of Johansson et al. (2021). They resemble the four types of resources and vulnerabilities (understood as an agency gap between VLGs and the ability to reach them) of older adults who have migrated to Sweden. The four cases are as follows. (1) Yara, whose family wants to care for her but cannot afford the loss of income it would involve. Yara is an example of the first type outlined above. (2) Hannah, who wants support from her social network to keep in touch with them, but is not sure how that kind of support can be arranged. Hannah is an example of the second type outlined above. (3) Ahmet, who has had a stroke and was previously active in ethnic organizations that he claims are suitable providers of culturally sensitive care. Ahmet is an example of the third type outlined above. (4) Rima, who would like for her care to be provided by her family, but whose family does not want to arrange it. Rima is an example of the fourth type outlined above.

See Table 1 for information on the different situations of Yara, Hannah, Ahmet and Rima.

**Table 1:** Characteristics of the four vignettes.

	Yara	Hannah	Ahmet	Rima
Age	74	75	66	70
Country of origin	Iran	Iraq	Turkey	Syria
Residing in Sweden since	Late 1990s	2000	approx. 1980	2016
Somatic difficulties	Edema, dizziness, fatigue	Osteoarthritis, emerging depression	Speech difficulties and dizziness due to stroke. Depression	Depression, anxiety, fractured arm
Help today	Home care: cleaning and cooking, applying ointments and hygiene. Family: compresson socks, reminding to take medicines	Home care: prepared meal boxes. Neighbors: help out with everything else	Home care: home chores, cooking and hygiene	Home care: safety alarm, everyday chores
Family relationships	Widow. Geographically close to four children and their families	Widow. Child and grandkids 20 km away. Family in Iraq	No family	Husband and son in Sweden. Rest of family scattered
Social network	Was previously the family hub, keeping track of people and organizing gatherings. Today she wants to continue to be an important part of the family, but has a hard time finding a new such role	Friends and neighbors	Active in a local cultural association for Kurds	Husband, son, daughter in-law
Income	SEK 10,000 / month (guaranteed pension). Children help with things that income cannot cover	Guaranteed pension and donations from family in Iraq	Sufficient from income pension	No information
Personal interests	Reading, listening to the radio, going for walks, helping family members	Gardening, social activities	Clubs and associations, gardening	Television

Each vignette was followed by a battery of 10 questions intended to direct the informant’s attention to the basic ideas of the WTH. The questions tap into the professionals’ attitude toward the needs of the individual, what information they pick up on as important in the case, what functions at the workplace need to be activated to support the individual, and what (if anything) they want to do for the individual that they cannot do today. They are as follows. (1) What in the vignette do you perceive as especially important for [name] to live a good life? (2) Based on the information in the vignette, do you think [name] has the resources to live up to his/her expectations about what good ageing entails? (3) What do you think [name] needs? (4) In your professional opinion, what interventions do you think [name] needs? (5) What additional information about the person do you think is important to enable you to do a good job? (6) You have been informed that [name] has certain goals that are important for his/her well-being. How can you use that information in your work? (7) In what ways can you as a professional make use of the information about how [name] wants the important goals to be achieved? (8) Who outside of your organization can you use as a support to do a good job with [name]. If any, how?

The questions are designed to stimulate the respondents to think about VLGs in terms of the welfare theory of health and about the resources that the persons in the vignettes have to reach their goals, which enables the adoption of a theoretical approach to the problem of cultural sensitivity and othering. The questions were answered in writing and the informants had unlimited space to write their responses. Prior to receiving the cases, the informants were given a short introduction with information about the study and instructions, followed by nine questions on the informants’ backgrounds.

### 2.3. Procedure and research ethics

The informants were contacted by e-mail with information about the study, their right to withdraw at any time without consequences, and their right to information about how their answers will be used and stored by the research team. After agreeing to participate, the informants received the vignettes and battery of questions as a digital survey with unlimited room for writing their answers. After finishing the survey, the informants sent their responses to the researchers by e-mail. The study was approved by the regional ethics committee in Uppsala (Dnr. 2018/279).

### 2.4. Analysis

The answers were analyzed using thematic analysis as outlined by Braun and Clarke (2006) with emphasis on the specific research questions of this study. The material was read several times to get oriented about the nature of the data. Initial codes were formulated and grouped into themes of commonality. The themes were reviewed, defined and named. Finally, the results were configured with concepts of the WTH and formatted into a written report. Informants’ names have been changed.

### 2.5. Validity

The vignettes are based on previous research, which found four types of agency gaps (Johansson et al., 2021) based on the welfare theory of health (Nordenfelt, 1993). Each vignette represents one of the four types of agency gaps. Using vignettes based on previous research strengthens a study’s validity (see Jergeby, 1999). To further strengthen validity, the themes were continuously read in relation to the data set to evaluate them as accurately reflecting the meaning of the data set, as described by Braun and Clarke (2006). Furthermore, the report was written in a way that provides plenty of vivid data extracts to demonstrate the prevalence of the reported themes (Braun and Clarke, 2006). The instrument’s validity was also enhanced by letting a test person read it and comment on ambiguities, after which revisions were made. More measures to improve validity were having two other researchers check and review the analysis of the empirical content and discuss the common findings. Finally, the entire manuscript has been reviewed, both methodologically and analytically, by fellow social work researchers during a review seminar.

## 3. Results

The battery of questions based on the holistic view of the WTH helped the assistant nurses isolate/identify the vital life goals of the people in the vignettes, assess their opportunities to reach the goals, and plan interventions to make the VLGs reachable. Also, the questions seem to have helped the assistant nurses to apply cultural sensitivity in their interventions, mostly based on individual preferences rather than fixed ideas about cultural characteristics, though with some exceptions. The presentation of results is organized around the research questions.

### 3.1. Research question 1

How do professionals perceive the vulnerabilities of older adults? (understood as an agency gap between VLGs and the ability to reach them).

We found three themes of vital life goals that the informants identified in the vignettes: (1) VLGs related to social networks, (2) VLGs related to activity, and (3) VLGs related to a feeling of safety. These indicated that the informants identify personal VLGs in the different cases, but also that as agency gaps grow wider in the vignettes, the informants find it increasingly difficult to identify VLGs to work with.

#### 3.1.1. Vignette 1, Yara

Yara’s case describes a woman who came to Sweden from Iran in the late 1990s in her 40s. She never firmly established herself in the labor market due to extended family responsibilities. She is portrayed in the vignettes as a person whose role in the family is very important, both to her and to others, and who is sad because she is slowly losing that role, as her children have become adults and her husband has passed away. Yara does not trust the municipality-provided care, mainly because of the risk of miscommunication, and wants her children to provide her care, but the children cannot afford to cut back their working hours. All the professionals recognize the agency gap manifested by Yara’s wish to keep a role that is gradually slipping away. They emphasize the importance for Yara of keeping her family relations and role within the family, which is even thought of as the main source of joy in her life: “She needs to find meaning in life and feel needed somehow” (IP11). The informants also acknowledge Yara’s lack of trust in the municipal elderly care and reformulate that mistrust into a need to feel safe in the caring situation. That feeling of safety is best achieved with tools for good communication. For example, this can be done by having a small workforce around Yara who can learn her ways and gain her trust. “//… from the home care service one / or two contact staff who often come to her, so they can build trust and understanding… //” (IP6).

Good communication is described as a way to gain Yara’s trust, so the home care professionals can help her feel safe. Thus, being able to communicate with a helper is important mainly for improving the care situation (doing a better job), rather than for the comfort of safety in itself. “As a professional, I believe that Yara needs to feel safe at home with the help she receives from us. For example, more often sending staff who speak Persian” (IP2). Regarding activity, walks are mainly identified as important for Yara. “Yara likes walks, so I can plan more walks during the day” (IP2).

#### 3.1.2. Vignette 2, Hannah

Hannah’s case describes a 75-year-old woman who migrated to Sweden in the early 2000s. Hannah has been a widow for a few years, and has a strong social network of neighbors and friends. She has not needed any interventions from municipal elderly care, since the network helps her with small things. Hannah’s need for help has now increased, and she realizes that the help she gets from her network will not be sufficient in the long run. Hannah worries that she might lose contact with her social network if the municipality takes on greater responsibility for her care.

The informants identified the relationship between Hannah and her friends and family as the most important thing for her, unlike Yara where the emphasis is on role preservation. For Hannah, the focus is mainly on protecting her against isolation by helping her stay in contact with her social network “…//not to isolate herself… //” (IP1), “Connect with her friends… //” (IP2). Engaging municipal care interventions is a way to strike a balance between receiving help from the municipality and from the social network, because she needs to “continue to have a social life with neighbors and family but accept that she needs help from the home service to be able to spend time with family and neighbors without being too much of a burden for them” (IP15). Here, IP15 wants to negotiate with Hannah to make her realize that she can keep her social relations if the municipal care organization can take over some of the work in some form of co-operation between the two. Regarding activity, household chores and leisure activities are mainly advocated “//…to do the chores and leisure activities she has done before” (IP8). To help Hannah reach her VLGs, the professionals emphasize the need to establish a relationship with Hannah to gain her trust, “Can talk to her a bit and create trust in the municipality’s staff” (IP1).

#### 3.1.3. Vignette 3, Ahmet

Ahmet’s case describes a 66-year-old man who recently suffered a stroke. Ahmet has no children but has previously been very active in the local Kurdish cultural association, where he has pushed for the need for culturally sensitive elderly care to be performed by NGOs and suggested that his cultural association could be a such provider. This is because, according to the association, a helper needs to understand certain culturally shaped preferences and be able to converse about what is important for, in this case, Kurds.

The professionals acknowledge as a VLG Ahmet’s need to stay active in the organizations that are important to him, and they try to bridge the agency gap of his being unable to receive the care that he advocated for before the stroke. IP 7 states, “Access to association activities, language and people from their culture” is important.

Culturally specific VLGs are also acknowledged in the form of sharing food preferences with care personnel. IP9 stated that “//… especially when it comes to cooking, someone who knows something about Kurdish food, so that he can feel that bit is familiar.” Food preferences are also presented as activity related VLGs “//… Being able to maintain their cultural preferences with friends and food” (IP6). In contrast to Yara and Hannah, the professionals did not identify any VLGs related to a feeling of safety in the Ahmet vignette.

#### 3.1.4. Vignette 4, Rima

The last vignette described Rima, a 70-year-old woman who recently migrated to Sweden from Syria due to the civil war. Rima has a husband and son in Sweden, and the rest of her family are geographically scattered around the world. Before the flight to safety, Rima had a very clear picture of her ideal ageing. She wanted a multi-generational living situation, and to be cared for by her family. Now, that vision is no longer possible, since the care regime in Sweden is not arranged in that way. In addition, her son’s partner is against generational care arrangements. Today Rima has some municipal care interventions, but is distressed about being unable to communicate with the helpers and establish a relationship with them. Rima’s vignette is the one that is based on the most vulnerable situation identified in the work of Johansson et al. (2021). This is reflected in the professionals’ struggle to find meaningful VLGs in the case. No activity- or safety-related VLGs were recognized, only socially related ones. Rima is portrayed as the person with the most acute agency gap. Socially, the professionals emphasize the need for communication with peers with a shared language, “Getting in touch with other people who speak the same language” (IP8), but also to engage social networks around Rima, “She must have a social network that works around her […] needs contact persons who can get her to go out and meet others until she can go to meeting places” (IP10).

### 3.2. Research question 2

The second research question directs attention to how the professionals perceive their own scope of action in working with VLGs as a tool. This is expressed differently across the vignettes.

In general, three themes are described as important when the professionals try to mitigate the agency gap. First is the need to understand how the person wants their VLGs to be achieved. The second is to establish a trusting relationship and gain a deeper understanding of the person’s preferences; they need to get to know the person. Thirdly, there is a need to extract and concentrate the information so that the care organization can work effectively with the person.

#### 3.2.1. Vignette 1, Yara

To mitigate the threat of losing the ability to realize Yara’s VLGs, the professionals first direct their attention to understanding her practical needs. This is expressed by “What help she needs //…” (IP12), what resources she has to reach her VLGs on her own, “//… what she can do herself” (IP11), and how she’d like to be treated, “how Yara wants to be treated” (IP12). In the vignette, Yara’s family was described as willing to help out with care chores, but not having the opportunity to take time off from work to the extent that would be required to be part of Yara’s care. Nevertheless, the professionals try to negotiate ways to incorporate the family into Yara’s care in order to realize her VLGs, “//…when the family has time to be there, you can compromise” (IP13). The informants also describe a need for negotiation around how the information Yara provides should be interpreted. In cases of ambiguity, they request an outside source for interpretation beside Yara herself, “…//Know who I can contact for questions and clarifications” (IP16).

To reach a deeper understanding of Yara’s preferences, the professionals describe a need to know more about her life in Syria and her leisure activity apart from family life, “What interests she has had besides her family…//” (IP8).

That information is later provided when the professionals try to learn about Yara’s VLGs and what they need to help her reach her goals, “… // show interest in the goals and supporting her in goal fulfillment… //” (IP6). Finally, the goals also need to be well-anchored within the care organization, so that all workers can work cooperatively to achieve them: “Then you have these goals to work toward in collaboration with the team (IP5).” The professionals express that the information gathered from Yara must be distributed and used across their care organization, preferably through a so-called implementation plan, “…// you write her implementation plan, which you then work by and follow up regularly” (IP15).

#### 3.2.2. Vignette 2, Hannah

Hannah expressed worries about losing contact with her social network if municipal elderly care took over the help she has been getting from friends and neighbors.

The professionals describe an agency gap in Hannah’s lack of trust in municipal care givers, even though the presence or absence of trust was not a specific issue in the vignette. Creating a trusting relationship is seen as a doorway to motivation: “I can talk to her a bit and create trust in the municipality’s staff” (IP1), “What I can do is talk to her and motivate her to exercise help at home as well as other assistance that she needs…//” (18). A plausible response to the issue of Hannah’s depression, which is affected by the anxiety she feels about her situation, is to “get to know Hannah as a person to help her see opportunities and joy in everyday life” (16).

Professionals also express a need to document Hannah’s VLGs to try and find ways to realize them, “finding strategies to work toward those goals” (IP7), which indicates a need for more information about personal preferences. It is also important to establish knowledge of Hannah’s VLGs within the organization, preferably in the so-called implementation plan, a joint document concerning a person’s care goals and preferences that are important for carers to know about: “we work with Hannah according to the goals; everyone follows the implementation plan” (IP3).

#### 3.2.3. Vignette 3, Ahmet

Ahmet has previously expressed ideas about integrating care into the activities of local cultural associations to achieve better cultural sensitivity. In Ahmet’s case, the informants do not acknowledge his advocacy for culturally sensitive care, but direct attention to Ahmet as a person. IP6 expresses this by mentioning the importance of “getting to know Ahmet as a person and showing interest in his story.” Understanding Ahmet as a person, rather than just a bearer of a certain culture, is not only a way to access his personal cultural preferences, it is a way to gain further understanding of his wishes: “the more you know about the person’s wishes and what they want and where they want to go in this life, the more it helps our work… //” (IP7). As a way to improve Ahmet’s interaction with his social network and need for cultural stimulation, the professionals see a possibility to help Ahmet stay in contact with his Kurdish association. IP20 expresses this as follows: “I can try to get in touch with the associations he has been part of and try to get them to come to Ahmet.” As with the previous vignettes of Yara and Hannah, the information, including about somatic issues, will need to be conveyed to the care organization, preferably along with the implementation plan.

#### 3.2.4. Vignette 4, Rima

Rima, the case with the most acute form of agency gap, wanted to be cared for by her family in a multi-generational living situation. However, her family is scattered, and those who are geographically near her do not want that kind of care arrangement. The professionals struggle to find goals to work with, and their response is thus to try and find out more details about her VLGs, “what she wants and her goals” (IP1). Initially, the professionals limit themselves to learning about Rima as a person, “what has Rima liked to do… //” (IP4), and her life in Aleppo, “What was Rima’s life like in Syria? Did she work? What were her interests and what did she like to do? Were they happy? What food did they eat?” (IP8). As in previous cases, the professionals express a need to establish a relationship: “I need to get to know Rima’s personality in order to treat her in a rehabilitative way” (IP15). Since the informants struggle to identify VLGs, and the ones that are expressed (generational care) are seemingly impossible to reach, the informants negotiate Rima’s VLGs. IP16 elaborates on how she wants to negotiate the VLGs “by helping Rima see if the goals are achievable, set interim goals or rewrite them into realistic ones.”

When the VLGs have been documented, they can be used by professionals as motivation for Rima to change: “Use the goals as a carrot to motivate yourself to challenge yourself and try new things” (IP15). Rima’s case description does not explicitly state that she wants anything to change, but it does mention that she is in a stressful situation, one that the professionals want to remedy.

As in the previous cases, it is important to anchor the VLGs among professionals within the organization, “I try to talk to colleagues who are around her to make sure that the same staff come as often as possible, so everyone knows what she has been through” (IP10). As part of their scope of action, the professionals have and use tools to mitigate stressful situations, for example motivational interviewing (MI): “By helping her work toward the goals, I make use of MI” (IP8).

## 4. Discussion

The welfare theory of health (WTH) is a theory that departs from the notion that what is important for an individual to experience a minimal level of happiness can be used as an individual state that a body must be able to reach in a state of health (Nordenfelt, 1993). Previous research (Johansson et al., 2021, 2022) has suggested that the threshold, known as vital life goals (VLGs), can be used to guide what cultural sensitivity should entail to avoid othering while staying sensitive to the cultural values that are important for a person. And that WTH can help reveal agency gaps that occur when culturally rooted expectations of ageing are out of reach due to migration. This study aimed to illuminate how the WTH can be used by professionals within elderly care as a foundation for managing the agency gap in achieving culturally sensitive care. The results show that when armed with questions regarding a person’s goals VLGs (see Nordenfelt, 1993), the assistant nurses presented nuanced ideas about how to approach the vulnerability associated with experiencing an agency gap between VLGs and the resources needed to achieve them. The socially themed VLGs are acknowledged in all vignettes in relation to older adults’ restricted ability to reach the VLGs, while activity and feelings of safety are not described as frequently and in such detail. Notably, as the vignettes changed and the vulnerability due to agency gaps increased, the assistant nurses found it increasingly hard to find VLGs to work with. In two of the cases, Hannah and Yara, the professionals describe a search for VLGs that ends up in a negotiation to change the VLGs into something that is achievable by the professionals. The vulnerability of the older adults who experience agency gaps as described in the vignettes becomes twofold, comprising both the agency gap itself and the difficulties that professionals find in working with and mitigating the agency gaps.

The professionals describe an intention to learn about the older adults’ VLGs and to gain a deeper understanding of their VLGs by learning about their lives. This is in line with the cultural humility described by Tervalon and Murray-García (1998) where patient-focused interviews and self-reflexivity are central to decision making where personal preferences should be guiding interventions and not care professionals perceived ideas about fixed social categories. The kind of othering in elder care that Torres (2006) has warned about can lead to stereotyping older adults with immigrant backgrounds. Focusing on personal preferences and planning the work accordingly are an expression of an individual approach to cultural sensitivity, which Johansson et al. (2021) describe as a way of safeguarding against othering. On the other hand, when the VLGs are hard to achieve, as with the vignettes of Rima and Hannah, the professionals focus on fixed factors rather than personal preferences to understand the VLGs. These results align with Johansson et al.’s (2022) study where professionals in decision-making positions described a wish to base decisions on individual preferences, but began focusing on fixed cultural factors as the older adults’ needs became more complex. Thus, it is argued that cultural sensitivity supported by the WTH is a viable path forward to balance between the othering risk (see Torres, 2006) and individual preferences (see Johansson et al., 2021). Professionals tend to gravitate toward individual preferences (i.e., VLGs), but because of unknown factors, they seem to fall back on sources other than the individual, like family members and textbook knowledge, to help them interpret the personal preferences. Thus, it is reasonable to use the WTH along with routines to examine where the information is gathered to safeguard against using sources that were not intended when deciding on interventions.

### 4.1. Strengths and limitations

The total number of participants in this study is limited, which affects the possibility to generalize the results. However, given the qualitative approach employed, generalization has never been the aim of this study. The qualitative research approach rather aims to tap into the lived experience of the informants, to “see the world with the eyes of the other” (Larsson, 2005, pp. 92), which is made possible with the vignette method (Brunnberg and Kullberg, 2013). Instead, the results are strengthened by their alignment with results from previous empirical studies, as well as with previous theoretical and philosophical publications on approaches to health and happiness (Nordenfelt, 1993). However, the results differ from those of previous studies (Lill, 2017), showing that ethnic relations impact how care work is organized and care needs are interpreted (Söderman and Rosendahl, 2016). The vignette technique is known for its suitability for detecting differences in attitudes and approaches regarding similar cases (Wilks, 2004; Brunnberg and Kullberg, 2013). In this study, the vignette technique has enabled a comparison of how professionals understand the different agency gaps. Because the results are limited to a small group of assistant nurses, further studies will be needed to determine how the welfare theory of health can be promoted among other professionals, for example organizational administrators and case managers.

## 5. Conclusion

The study reveals that the assistant nurses in this study primarily adapted to the individual preferences of older adults, rather than fixed parameters based on cultural traits, but that they fell back on alternative sources when the complexity of the agency gaps increased. The professionals demonstrated that they can use the WTH (as framed in this study) as a means of addressing cultural sensitivity, thereby limiting the risk of othering and stereotyping older adults with immigrant background. The theory thus needs to be used with caution and preferably with a strategy for maintaining awareness of what sources are used when working to close the agency gap. The study also shows that the agency gap described by Johansson et al. (2021) is exacerbated, as professionals find it increasingly difficult to identify VLGs to work with older adults with an acute agency gap. Finally, the study shows that the underlying theoretical foundation (the welfare theory of health) is viable from the perspective of assistant nurses, which underlines the relevance of the WTH in culturally sensitive elderly care.

## Data availability statement

The datasets presented in this article are not readily available because the informants have accepted to participate in this study under the premises that their data will not be shared with anyone outside the research team. Requests to access the datasets should be directed to carl.johansson@mdu.se.

## Ethics statement

The studies involving humans were approved by the Regional ethics committee in Uppsala. The studies were conducted in accordance with the local legislation and institutional requirements. The participants provided their written informed consent to participate in this study.

## Author contributions

CJ contributed to the conception and design of the study, data collection, analysis of data, and the process of writing and revising the manuscript. DL contributed to the design of the study, analysis of data and the process of writing and revising the manuscript. IA and L-KG contributed to the conception and design of the study, analysis of data, and revising the manuscript. All authors contributed to the article and approved the submitted version.

## Funding

This work was financially supported by the Forte: Swedish Research Council for Health, Working Life and Welfare (2017-00031) and Mälardalen University, Sweden.

## Conflict of interest

The authors declare that the research was conducted in the absence of any commercial or financial relationships that could be construed as a potential conflict of interest.

## Publisher’s note

All claims expressed in this article are solely those of the authors and do not necessarily represent those of their affiliated organizations, or those of the publisher, the editors and the reviewers. Any product that may be evaluated in this article, or claim that may be made by its manufacturer, is not guaranteed or endorsed by the publisher.
